# Biomechanical analysis of different lifting speeds when using an active exoskeleton

**DOI:** 10.3389/fbioe.2025.1685634

**Published:** 2025-11-11

**Authors:** Dominik Mayer, Tobias Siebert, Jens Hasenmaier, Norman Stutzig

**Affiliations:** 1 Motion and Exercise Science, University of Stuttgart, Stuttgart, Germany; 2 Stuttgart Center for Simulation Science, University of Stuttgart, Stuttgart, Germany

**Keywords:** exoskeleton, active, musculoskeletal disorders, lower back, apogee, electromyography, muscle activity, movement velocity

## Abstract

**Introduction:**

Musculoskeletal disorders (MSDs), especially lower back pain, are common consequences of repetitive and long-term mechanical stress. Exoskeletons offer a promising approach to reduce this stress by supporting the wearer during physical labour. This study investigated the effect of an active exoskeleton (Apogee) on muscle activation and joint kinematics during load lifting at different lifting speeds and exoskeleton support levels.

**Methods:**

Sixteen healthy young adults (8 male, 8 female) lifted a 15 kg box at two lifting speeds (9 and 12 lifting cycles/min) and four support levels: 1) without exoskeleton, 2) exoskeleton in passive mode, 3) 50% support and 20% counterforce, 4) 100% support and 60% counterforce. Muscle activity was measured in the M. erector spinae (MES), M. biceps femoris (MBF) and M. vastus medialis (MVM) using EMG. Furthermore, joint range of motion (ROM) in the ankle, knee and hip were analysed using 3D motion capture.

**Results:**

Faster lifting significantly (p < 0.05) increased MBF (by 4.0% ± 1.5% maximum voluntary contraction, MVC) and MVM (1.6% ± 0.7% MVC) activity, while MES remained unaffected. The highest support level led to a significant decrease in MES and MBF activity by about 22.3% MVC and 10.6% MVC, respectively, as well as a small increase in hip joint ROM by 6° compared to lifting without exoskeleton support. There was no interaction between the level of support and lifting speed.

**Discussion:**

The decrease in MES activity of 22.3% MVC with full support suggests a potent reduction in spinal load. MBF activity increased less with higher speeds when support was applied. The MVM showed low and stable activity across all conditions. These findings suggest that the active exoskeleton Apogee provides support regardless of lifting speed and may help prevent MSDs in occupational settings. Users can adjust support levels based on task requirements and personal comfort.

## 1 Introduction

Musculoskeletal disorders (MSDs) are among the most prevalent work-related health conditions and have a multifactorial origin (Schneider et al., 2010). They encompass injuries and impairments affecting muscles, bones, nerves, tendons, ligaments, cartilage and intervertebral discs (Da Costa and Vieira, 2010). Within occupational settings, these conditions are referred to as work-related MSDs. There is evidence of a causal relationship between physical stress and the development of work-related MSDs (Andersen et al., 1997). Lower back disorders, in particular, have a high prevalence and represent the most common form of MSDs globally (Holzgreve et al., 2023). In the EU, for example, 20% of workers deal with one or more MSD, with 43% of these cases involving lower back problems. Risk factors include repeated lifting of heavy objects and maintaining uncomfortable or painful postures in the working environment (European Agency for Safety and Health at Work, 2019). MSDs result in longer absences from work, leading to high costs for both the companies and the whole industry. It is estimated that 30%–40% of these absences could be avoided through preventive measures and supportive workplace interventions (Thiehoff, 2002).

Exoskeletons represent a promising solution to reducing the muscle stress during the lifting of heavy loads. They are designed to mitigate physical stress on workers and prevent incapacity for work (Schick, 2018). Based on their support mechanisms, exoskeletons are classified as either active or passive systems. Active exoskeletons assist the lifting motion using electric motors, while passive exoskeletons provide support through mechanical elements such as elastic springs (Crea et al., 2021).

In a review by Kermavnar et al. (2021) on the effects of exoskeletons on body loading, 27 of the 33 included studies assessed the muscle activity of the back and hip extensors, such as the m. erector spinae (MES). On average, the studies reported a reduction in MES activity of 25% with active exoskeletons and 18% with passive exoskeletons. These findings are supported by recent studies. Huysamen et al. (2018) reported reductions in muscle activity of the MES and m. biceps femoris (MBF) of 15% and 5%, respectively, when lifting a 15 kg box. Koopman et al. (2019) found a 13.3% reduction in MES muscle activity, coupled with a 25% decrease in task completion time. Recently, there has been growing interest in actively powered, back supporting exoskeletons (Reimeir et al., 2023; Walter et al., 2023). Both studies investigated the “Cray X″ exoskeleton (German Bionic Systems GmbH, Augsburg, Germany). It allows users to select different levels of support for the task at hand. Walter et al. (2023) found that an increase in support level was associated with a reduction in MES activity, with a relative decrease of 22% when comparing lifting without the active exoskeleton to lifting with maximum support. Reimeir et al. (2023) observed a 35% reduction in MES activity when lifting a 15 kg box, together with an increase of 0.34–0.35 s in task completion time. It can be concluded that active exoskeletons support the lower back when lifting heavy weights.

However, the type of support might depend on the speed of movement, since both the muscle force (Hill, 1938; Siebert et al., 2015) and the torque of the electric motor (Santiago et al., 2012) depend nonlinearly on the contraction speed and the rotational speed respectively. Furthermore, the effectiveness of the exoskeleton depends on its regulation control, i.e., on how quickly the supporting torque is built up.

In everyday working tasks, varying lifting speeds are common, making it necessary to understand how speed influences muscle activation. In several studies, no prescribed lifting speed was given, and either the speed was not recorded (Huysamen et al., 2018; Walter et al., 2023) or the speed was recorded, but not standardized (Koopman et al., 2019; Reimeir et al., 2023). The effect of lifting speed on the neuromuscular activation of key muscles such as the MES, MBF and m. vastus medialis (MVM) has not yet been thoroughly investigated. The MES and MBF play crucial roles in hip extension and trunk stabilization during lifting, while the MVM contributes to knee extension and patellar stabilization (Hall, 2011).

The aim of the study is to analyse the neuromuscular activity of the MES, MBF and MVM and the range of motion (ROM) of the hip, knee and ankle joints at two different lifting speeds (45 bpm and 60 bpm). The slower pace (45 bpm) represents a natural working pace, whereas 60 bpm simulates a more hectic work environment. Both speeds were tested with (active vs. passive) and without exoskeleton assistance, using a stoop lifting technique. It is hypothesized that there is an interaction between lifting speed and support level with respect to muscle activation when using an active exoskeleton, indicating that both factors influence each other. The main contribution of this study is to determine whether the lifting speed has an influence on the effectiveness (in terms of altered muscle activation) of an exoskeleton. The influence on effectiveness is examined at different support levels of the exoskeleton.

## 2 Methods

### 2.1 Participants

19 young and healthy adults participated in the study. All participants reported being physically active, with about 20% exercising less than 3 hours per week and 80% exercising more than 3 hours per week. Three subjects had to be excluded due to technical issues: one because of EMG recording failure, one due to problems in kinematic recording and one due to a software malfunction of the exoskeleton during the second part of the experiment. Therefore 16 subjects (eight male, eight female; age: 21.8 ± 2.6 years; weight: 69.8 ± 10.0 kg; height: 176 ± 8 cm) were included in the evaluation. None of them had prior experience with exoskeletons. The subjects were informed about possible risks of the experiment and gave their written consent. The study was approved by the University of Stuttgart Ethics Committee (AZ. 24-034) and was conducted in accordance with the latest declaration of Helsinki.

### 2.2 Exoskeleton

**FIGURE 1:**
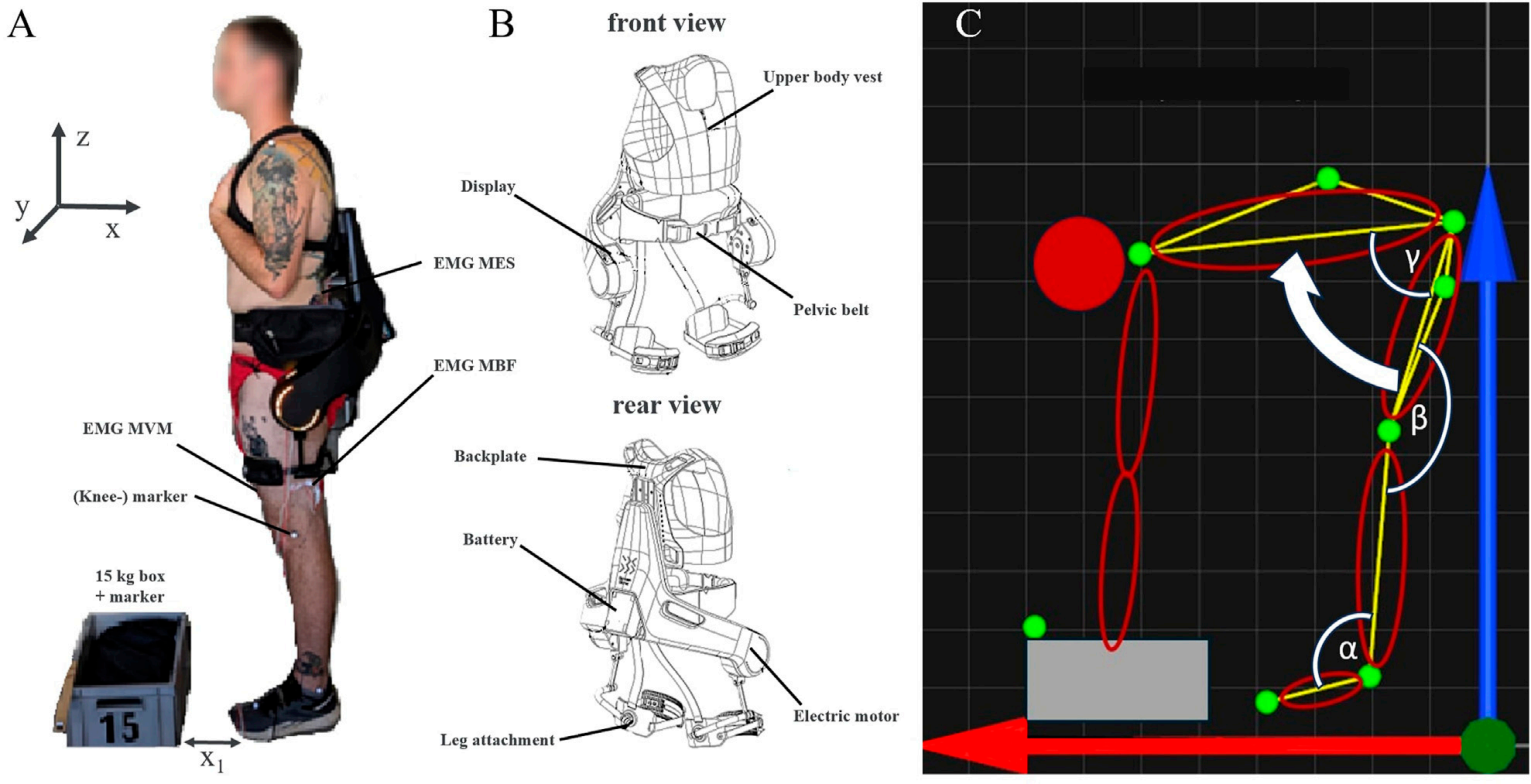
**(A)** Subject wearing the Apogee exoskeleton in front of the 15 kg box. x_1_ represents the horizontal distance between the participant and the load (about 15 cm). The box has the dimensions (40 × 30 × 22 cm) and lies with its short side in the direction of the sagittal axis (x) of the body. The box was grasped by the side handles with both hands. No gloves were worn. The initial and final height of the load were 0 cm and 64.2 ± 4.1 cm, respectively. Both feet were parallel to each other with a distance of about 20 cm. The reflective markers (white spheres) are labelled as an example for the knee joint. The positions of the EMG electrodes are indicated for the MES, MBF and MVM. **(B)** Schematic illustration of the Exoskeleton “Apogee” used in this study. **(C)** Schematic representation of the stoop-technique. The main movement is indicated by the white arrow. The green dots correspond to the positions of the optical markers. Red (x) and blue (z) arrows indicate the horizontal and vertical directions, respectively. The calculated joint angles are marked in white: α–ankle, β–knee, γ–hip.

In this study, the commercially available active exoskeleton Apogee (German Bionics Systems GmbH, Augsburg, Germany) was used (Figure 1). The Apogee is the successor of the Cray X model investigated by Walter et al. (2023) and provides enhanced support capabilities due to a more powerful electric motor and improved upper-body fixation systems. The device weighs approximately 8 kg and is worn like a hiking backpack. Most of the weight is distributed via a padded hip belt, resting mainly on the iliac crest. A chest harness and two leg attachments provide additional stabilization. The position and inclination of the user’s trunk is detected by inertial measurement units. This sensor data enables the device to recognize movement patterns and apply supportive torque to the hip joints during lifting. The exoskeleton offers to two types of support: counterforce and extension. Counterforce is applied during the downward bending phase, while extension support is provided during the upright return movement. Both support modalities can be adjusted in 10% increments, ranging from 0% to 100%.

### 2.3 Procedure

To investigate the impact of the exoskeleton and different levels of support at two lifting speeds (45 bpm vs. 60 bpm), the muscle activity of the MES, MBF and MVM was measured using EMG. Lifting speed was controlled using a metronome, each beat representing one of the five parts of the lifting motion: bending down, lifting the box, standing upright, setting the box down and returning to an upright position. Therefore, a lifting cycle consisted of five beats, resulting in nine and twelve lifting cycles per minute for 45 bpm and 60 bpm, respectively. The four different support levels were defined as follows: 1) without exoskeleton, 2) with exoskeleton in passive mode (no active support), 3) with active exoskeleton (50% support and 20% counterforce), and 4) with active exoskeleton (100% support and 60% counterforce).

**FIGURE 2:**
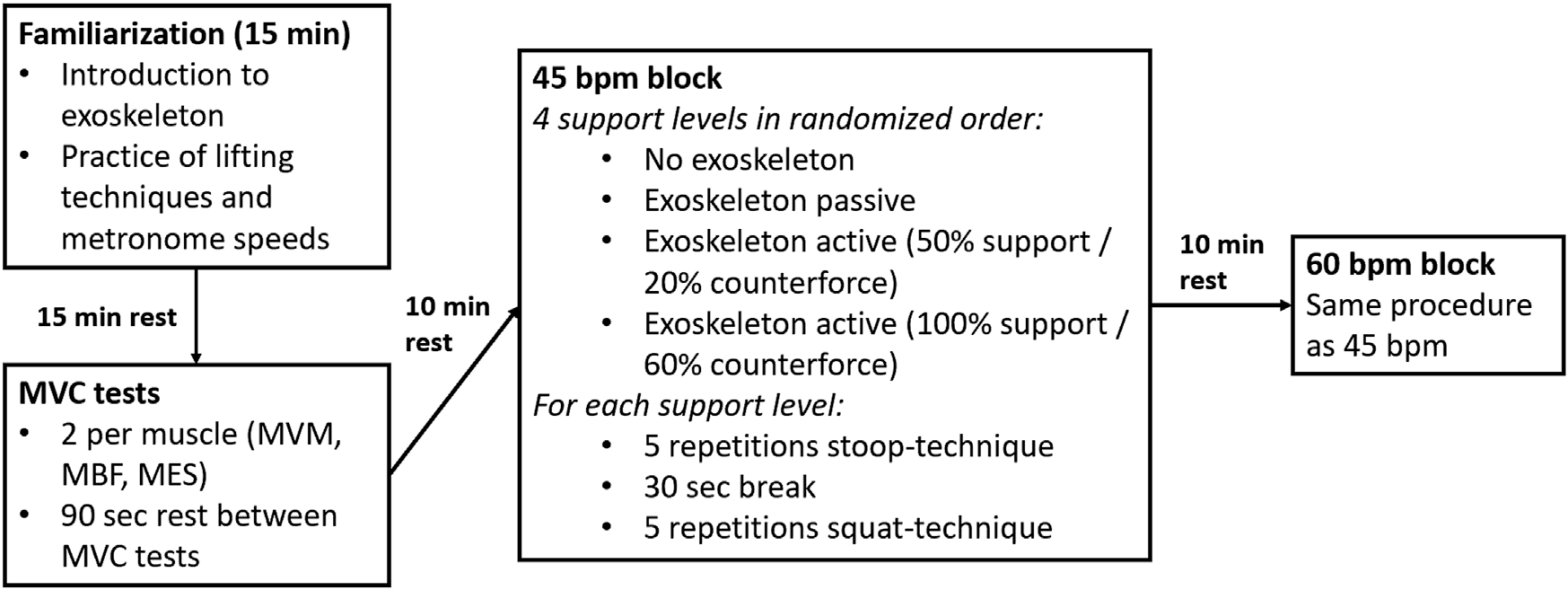
Schematic structure of the study procedure (MVC, maximal voluntary contraction; MVM, musculus vastus medialis; MBF, musculus biceps femoris; MES, musculus erector spinae; bpm, beats per minute).

The schematic structure of the study procedure can be seen in Figure 2. At the beginning of the experiment two maximum voluntary contraction (MVC) tests were conducted for each muscle (Gruber et al., 2009). Between the MVC measurements the subjects had a break of 90 s, after the MVC measurements a break of 10 min. Then, the subjects were instructed to lift a 15 kg box (40 × 30 × 22 cm) five times with each speed and level of support from the ground to standing upright. The chosen weight was in line with previous studies using similar experimental designs (Huysamen et al., 2018; Walter et al., 2023), ensuring comparability of the results. The stoop-technique was evaluated throughout this study, which is characterized by slightly bended or straight knees and a forward bend of the trunk (Figure 1). The order of the conditions was randomized but the subjects either started or ended the experiment without the exoskeleton. This ensured that there were no complications with the cable driven EMG device while putting the exoskeleton on or taking it off during the experiment. Each condition started with five repetitions using the stoop-technique. After a 30 s break, five repetitions using the squat technique were performed. A five-minute rest period was provided between each condition to minimize fatigue. After completing all conditions with 45 bpm, the subjects took a ten-minute break before repeating all conditions with 60 bpm. The lifting speed was controlled using a metronome, whereby each part of the movement (bending down, lifting the box, standing still with the box, bending down and placing box, straighten up) had to be done in one beat. To ensure correct handling of the exoskeleton and familiarize with the different lifting speeds and techniques, the subjects were introduced to the exoskeleton and had a 15-min familiarization session prior to the start of the experiment. Following this, the participants rested for 15-min to avoid fatigue effects.

### 2.4 Data recording

To measure muscle activity during lifting, bipolar surface EMG measurements were conducted on the following muscles of the left body side: M. erector spinae (m. longissimus), m. biceps femoris and m. vastus medialis (Figure 1). Prior to electrode placement, the skin was shaved, abraded with sandpaper and cleaned with alcohol to increase skin conductivity and signal quality (Hermens et al., 1999). The muscles were located according to SENIAM recommendations. For the MES, two self-adhesive EMG electrodes were placed with a 2 cm interelectrode distance on the MES approximately two fingers wide outside the spinous process of the first lumbar vertebra (L1). The MBF electrodes were placed midway between the ischial tuberosity and the lateral epicondyle of the tibia. For the MVM, electrodes were positioned at 80% of the distance between the anterior superior iliac spine and the knee joint space, oriented perpendicular to the line connecting both landmarks (Hermens et al., 1999). In addition, a reference electrode was placed on the medial malleolus. In general, electrode placement was not impeded by the Apogee exoskeleton. However, in a few cases, electrodes had to be shifted slightly upwards to avoid pressure from the attachment system. The EMG signals were recorded with a sample rate of 2 kHz (MP160, Biopac Systems, Goleta, United States), pre-amplified (2000), band-pass filtered (bandwidth 10–500 Hz), and stored on a computer.

For kinematic analysis, a 3D motion capture system with four infrared cameras (Qualisys AB, Gothenburg, Sweden) was used. Reflective markers were placed on the left side of the body at the following anatomical landmarks: Os metatarsale II, malleolus lateralis, lateral knee joint gap, trochanter major, on a line between trochanter major and knee joint gap, crista iliaca and one on a line between trochanter major and crista iliaca, plus an extra marker on the box to determine the start of the lifting phase (Figure 1A). Motion data was captured at 340 Hz using the Qualisys Track Manager 2024.2 (Qualisys AB, Gothenburg, Sweden).

### 2.5 Data processing

Only the period in which the box was lifted was analysed. Therefore, the analysed movement was defined from the onset of lifting until the box reached its highest point. The EMG and kinematic data were processed using Matlab® 2024b (The MathWorks Inc., Natick, Massachusetts, United States). The EMG data were fully rectified, smoothed with a 150 ms moving average and normalized to the MVC measurement for each muscle. Afterwards the maximum EMG values during each lifting trial were determined for each subject.

The kinematic data was used to define the starting and final position and to verify the execution of the stoop-technique. Due to the use of only four cameras, the 3D coordinates of the markers were reduced to the sagittal plane, which allowed a 2D vision on the relevant angles (Figure 1). To determine the influence of the exoskeleton on the ROM of the ankle, knee and hip joints during the stoop-technique, joint angles were calculated based on the orientation between specific markers. The ankle joint angle was defined as the angle between the line connecting the foot and ankle markers and the line between the ankle and knee markers. The knee joint angle was calculated from the angle between the line connecting the ankle and knee markers and the line between the knee and hip markers. The hip joint angle was determined based on the angle between the line connecting the knee and hip markers and the line between the hip marker and the marker placed on the crista iliaca. Due to the padded hip belt, the hip marker could not be recorded in the trials using the exoskeleton. For this reason, the location of the hip marker was calculated based on the relative location and distance between the knee and upper-thigh marker, based on a static reference trial at the beginning of the experiment.

### 2.6 Statistical analysis

The data are presented as means and standard errors (SE). All data were checked for normal distribution using the Kolmogorov–Smirnov test and met the criteria. A 2 (SPEED [45 bpm vs. 60 bpm]) X 4 (LEVEL OF SUPPORT [without Exo vs. with Exo (0/0%) vs. with Exo (50/20%) vs. with Exo (100/60%)]) repeated-measures analysis of variance (rANOVA) was conducted for the EMG and kinematic data. Mauchly test was used to confirm the condition of sphericity for the factor support level and for the interaction between speed and support. In cases where sphericity was violated (*p* < 0.05), a correction method was used depending on the Greenhouse-Geisser-Epsilon (ε): if ε > 0.75, the Hyun-Feldt correction was used, otherwise the Greenhouse Geisser correction was applied (Rasch et al., 2014). Effect size (Equation 1) was determined using partial eta squared ( $\eta_{P}^{2}$ ):

$$\eta_{P}^{2}=\frac{{SS}_{effect}}{{SS}_{effect}+{SS}_{error}}$$ (1)

where ${SS}_{effect}$ is the sum of squares of the interested effect and ${SS}_{error}$ the sum of squares of the error term of the interested effect. The effect sizes were classified as low ( $\left.\eta_{P}^{2}=0.01\right)$ , medium ( $\left.\eta_{P}^{2}=0.06\right)$ and large ( $\left.\eta_{P}^{2}=0.14\right)$ (Cohen, 1988).

When the rANOVA showed significant main effects or interactions, *post hoc* analyses were performed using the Bonferroni correction. The level of significance was set at *α* = 0.05. Results are reported as mean ± standard error of the mean (SEM). The statistical analysis was performed using SPSS (Version 29.0, IBM SPSS Statistics).

## 3 Results

### 3.1 Comparison of pooled data: Speed

**FIGURE 3:**
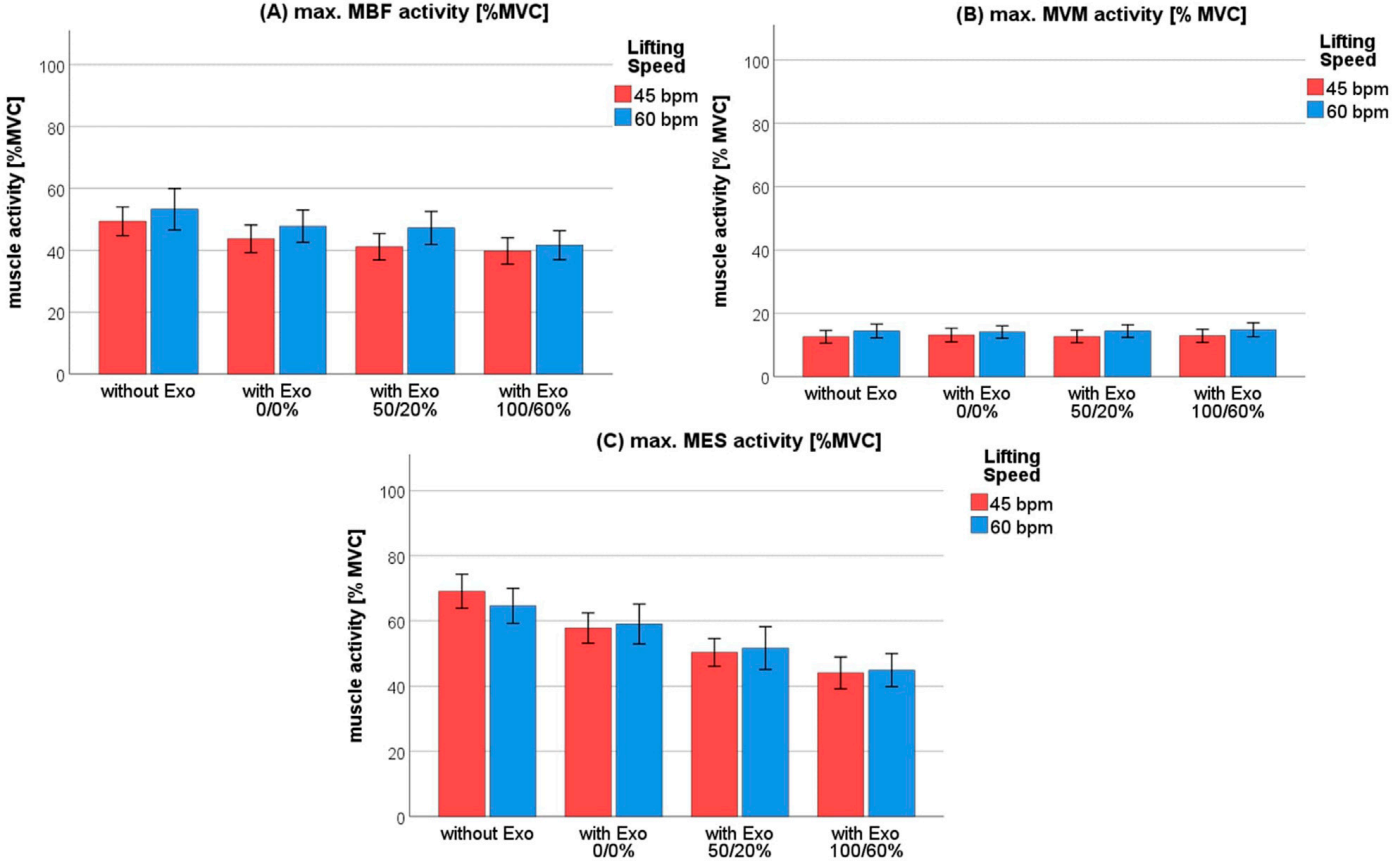
Mean muscle activity of the m. biceps femoris **(A)**, m. vastus medialis **(B)** and m. erector spinae **(C)** across four exoskeleton support levels when lifting at two different speeds: 45 bpm–red, 60 bpm–blue.

To investigate the differences in muscle activity and ROM between the two lifting speeds, the four support levels were pooled for 45 and 60 bpm separately. The rANOVA showed a significant effect of lifting speed on MBF activity, *F* (1,15) = 7.22, *p* = 0.017, $\eta_{P}^{2}$ = 0.325. Muscle activity increased by 4.0% ± 1.5% MVC from the slower speed (43.5% MVC, *SEM* = 4.3% MVC) to the faster speed (47.5% MVC, *SEM* = 5.4% MVC). Similarly, for the MVM the rANOVA showed a significant effect of lifting speed, *F* (1,15) = 5.9, *p* = 0.028, $\eta_{P}^{2}$ = 0.282. The muscle activity increased by 1.6% ± 0.7% MVC from 45 bpm (12.8% MVC, *SEM* = 2% MVC) to 60 bpm (14.4% MVC, *SEM* = 2% MVC) (Figure 3B). In contrast, no significant effects of lifting speed were observed for MES activity. Additionally, no significant effects of lifting speed were observed for the ROM of the hip, knee or ankle joints (Table 2).

### 3.2 Comparison of pooled data: level of support

To evaluate the effect of different support level, data from both lifting speeds were pooled. The rANOVA showed a significant influence of support level on the activity of the MBF, *F* (3,45) = 13.6, *p* < 0.001, $\eta_{P}^{2}$ = 0.476. As can be seen in Figure 3C, *post hoc* analysis showed a progressive reduction in MBF maximum muscle activity: significant differences were found between level 1) (51.3% MVC, *SEM* = 5.6% MVC) and 2) (45.7% MVC, *SEM* = 5.4% MVC) (*p* = 0.007), between level 1) and 3) (44.2% MVC, *SEM* = 4.8% MVC) (*p* = 0.01) and between level 1) and level 4) (40.7% MVC, *SEM* = 4.4% MVC). MES activity was also significantly affected by support level, *F* (1.92,28.8) = 28.8, *p* < 0.001, $\eta_{P}^{2}$ = 0.656, Greenhouse-Geisser corrected. Post-hoc analysis showed significant reductions in MES activity between level 1) (66.8% MVC, *SEM* = 5.0% MVC) and both level 3) (51.0% MVC, *SEM* = 5.2% MVC) and 4) (*M* = 44.5% MVC, *SEM* = 4.9% MVC) (*p* < 0.001), as well as a significant difference between level 1) (58.4% MVC, *SEM* = 5.2% MVC) and level 2) and 3) (*p* < 0.001). Lastly, MES activity differed significantly between level 3) and 4) (*p* < 0.018) (Figure 3C). No significant effect of support level was observed for MVM activity (Figure 3B).

The rANOVA showed a significant influence of support level on the ankle ROM (*F* (1.90, 28.5) = 4.35, *p* = 0.024, $\eta_{P}^{2}$ = 0.225, Greenhouse-Geisser corrected). Bonferroni-corrected *post hoc* tests showed a significant difference (*p* < 0.001) between level 3) (5.5°, *SEM* = 0.65°) and level 4) (6.7°, *SEM* = 0.7°) (Table 1). Similarly, the ROM of the hip got significantly affected by the level of support, *F* (1.87, 28.0) = 7.53, *p* = 0.03, $\eta_{P}^{2}$ = 0.334, Greenhouse-Geisser corrected. Post-hoc tests showed significant increases between the support level 1) without exoskeleton (85.5°, *SEM* = 2.09°) and level 4) with maximum support (91.5°, *SEM* = 1.7°) (*p* = 0.018), as well as between support level 2) (87.9°, *SEM* = 1.9°) and 4) (*p* = 0.004). No significant effect of support level was found for knee ROM (Table 2).

**TABLE 1:** Mean and standard error for the ROM in the ankle, knee and hip joint.

	Mean and standard error					
	Speed		Level of support			
	45 bpm	60 bpm	1	2	3	4
Ankle ROM (°)	5.8 ± 0.6	6.4 ± 0.7	6.6 ± 0.6	5.7 ± 0.7	5.5 ± 0.7	6.7 ± 0.7
Knee ROM (°)	26.2 ± 3.1	24.9 ± 3.2	25.2 ± 3.4	25.1 ± 2.9	25.2 ± 3.0	26.6 ± 3.3
Hip ROM (°)	88.3 ± 1.7	88.6 ± 1.9	85.5 ± 2.1	87.9 ± 1.9	88.8 ± 1.8	91.5 ± 1.7

**TABLE 2:** Pairwise comparisons using the Bonferroni-correction for the ROM in the ankle, knee and hip joint.

	Pairwise comparisons (Bonferroni-correction)						
	Speed	Level of support					
	45/60 bpm	1/2	1/3	1/4	2/3	2/4	3/4
Ankle ROM (°)	−0.7 ± 0.4 p = 0.129	1.0 ± 0.4, p = 0.256	1.1 ± 0.5, p = 0.343	−0.1 ± 0.5, p = 1.00	0.1 ± 0.4, p = 1.00	−1.1 ± 0.4, p = 0.057	−1.2 ± 0.2, p <0 .001***
Knee ROM (°)	1.3 ± 1.5, p = 0.398	0.1 ± 1.4, p = 1.00	−0.1 ± 2.0, p = 1.00	−1.5 ± 1.5, p = 1.00	−1.5 ± 1.0, p = 1.00	−1.6 ± 0.9, p = 0.519	−1.4 ± 1.0, p = 1.00
Hip ROM (°)	−0.3 ± 1.3 p = 0.820	−2.4 ± 1.6, p = 0.899	−3.3 ± 1.4, p = 0.179	−6.0 ± 1.7, p = 0.018*	−0.9 ± 0.7, p = 1.00	−3.6 ± 0.8, p = 0.004**	−2.7 ± 1.1, p = 0.178

Significance: *p < 0.05, **p < 0.01, ***p < 0.001.

### 3.3 Interaction effects: Speed and level of support

No significant interaction effects of lifting speed and support level were found for ROM of the hip, knee or ankle joints, nor for the muscle activity of MES, MBF or MVM (Figure 3).

## 4 Discussion

The aim of this study was to investigate the effects of different lifting conditions and speeds on muscle activity while using an active exoskeleton. First, increasing the lifting speed from 45 bpm to 60 bpm significantly affected thigh muscle activity (MBF, MVM), but not MES (Figure 3) and ROM (Table 2). Second, the level of support showed a significant effect on muscle activity for the MBF and MES but not for MVM (Figure 3). No interaction effects between lifting speed and support condition were observed for any variable. The absence of significant interaction effects indicates that users can choose the appropriate level of support for the task at hand, regardless of the working speed.

### 4.1 Comparison with literature

In this study, the observed relative reduction in MES muscle activity between lifting without an active exoskeleton and maximum support was 33%. This falls within the range reported by Kermavnar et al. (2021), who observed reductions between 5% and 48%, with an average of 25%. In contrast, the relative reduction of 21% in MBF muscle activity between lifting without an active exoskeleton and maximum support is higher than the reported 5% average in the literature (Kermavnar et al., 2021). One possible explanation for this discrepancy could be the execution of different lifting techniques (squat or stoop technique). The squat technique is characterised by high knee flexion and shifts the muscular activation toward knee extensors such as the MVM and reduces the relative contribution from hip extensors like the MBF (van der Have et al., 2019; Washmuth et al., 2022). Meanwhile the stoop technique is mainly initiated at the hip joint and therefore requires higher activation of the MBF (Hall, 2011). Since the studies included in the review did not analyse lifting kinematics, it is possible that the use of an exoskeleton caused a shift towards the squatting technique (Kermavnar et al., 2021). In general, differences in exoskeletons, lifting movement, lifting speed and box weight can influence muscle activation and are therefore potential causes of differences between studies.

Interestingly there were already differences in muscle activation for the MBF and MES between support level 1), lifting without exoskeleton, and support level 2), lifting in passive mode. The activity of the MBF and MES reduced by 5.6% MVC and 8.4% MVC, respectively, while the difference for the MES was not statistically significant (*p* = 0.083). This difference in muscle activity could be observed for 75% of the 16 participants. The decrease in MES activity may be caused from the structural elements of the Apogee exoskeleton. Van Poppel et al. (2000) described that even passive lumbar orthoses can influence the muscle activity, likely due to mechanical compression and restricted motion of the trunk. The padded hip belt and rigid back plate further may limit the spinal flexion, possibly leading to a decreased stabilizing demand on the MES during lifting thus reduced muscle activity. The absence of reduction in muscle activity of the remaining participants may be attributed to individual anthropometric differences, which can affect the fit and mechanical interaction between the exoskeleton and the user.

### 4.2 Effects of the exoskeleton on muscle function at different lifting speeds

As outlined in 4.1, the lifting motion with the stoop technique is mainly initiated at the hip joint. This is supported by the comparatively large ROM in the hip joint of approximately 90° (in comparison to 25° in knee ROM and 6° in ankle ROM) (Table 1). In the absence of exoskeleton assistance, the torques necessary for the motion at the hip joint are mainly produced by the hamstring muscles (e.g., MBF) and the gluteus muscle (not measured). Since the exoskeleton provides torque support at the hip joint, an expected reduction in MBF activity can be observed when the device is used. Without exoskeleton support, increased lifting speeds require higher torque at the hip joint, and thus higher muscle activity. This was also confirmed by our experiments without exoskeleton which showed an increased MBF activity by 3.9% MVC when lifting speed rose from 45 bpm to 60 bpm. With maximum exoskeleton support MBF activity only increased by 1.9% MVC under the same speed conditions (Figure 3A).

In the stoop lifting technique, the MVM is mainly responsible for stabilizing the knee joint, which is reflected by the relatively small ROM observed at the knee (Table 1). As can be seen in Figure 3B, MVM activity remained low at around 15% MVC and did not get affected by the use of the exoskeleton. The minor increase of 2% MVC at higher lifting speeds (from 45 to 60 bpm, Figure 3B) is likely due to the greater stabilization demands associated with faster movements.

The MES primarily stabilizes the spine and does not contribute to the torque generation at the hip joint in the stoop technique. The high MES activity of 66.8% MVC observed during lifting without the exoskeleton (Figure 3C) is likely due to the high stabilization demands and results in considerable mechanical stress of the spine. When using the exoskeleton at maximum support (level 4), MES activity was significantly reduced by 22.3% MVC, which might result in a reduced spinal load through exoskeleton use. As noted by Kermavnar et al. (2021), muscle activity is correlated with mechanical stress. Provided the movement pattern remains similar, a reduction in MES activity due to exoskeleton support translates to a reduction in mechanical stress. Given the minor increase in hip ROM (Tabe 1), it can be deduced that the active exoskeleton Apogee reduces effectively the mechanical stress of the lower back and further the development of work-related MSD (Coenen et al., 2014).

Reducing mechanical stress on the lower back could improve postural stability in neuromuscular diseases, especially in people with spinal cord injuries. Altered movement patterns in agonist-antagonist activation have been observed in patients with spinal cord injuries (Shan et al., 2025a). The results show that postural stability is impaired by uncoordinated lumbar and overactive thoracic neuromuscular control despite consistent speed. This leads to greater instability in balance control when sitting (Shan et al., 2025b). In addition to supporting movement, the use of exoskeletons could also contribute to increasing motion control and balance in patients with neuromuscular impairments. However, further research and exoskeleton adaptations to specific disease cases are needed.

### 4.3 Limitations

One limitation of the present study is the estimation of hip joint position based on marker placement. While necessary for calculating hip ROM, minor differences from actual joint angles are to be expected. Another limiting factor is the narrowing down on one lifting technique and a specific part of the lift. This limits the generalizability of the findings to varied manual handling tasks.

In addition, only a limited number of muscles were analysed. Although the hamstrings (e.g., MBF) were included as key hip extensors, the gluteus muscle groups likewise play an important role in generating hip extension torque. To generalize the findings of this research, studies should expand the included muscles to better understand the effect of exoskeleton usage on muscle activity and control.

Fatigue effects were not systematically assessed in this study. However, sufficient rest periods were provided between trials, to minimize potential influences of muscle fatigue on EMG activity. In future, the maximum M-wave could be used to monitor possible peripheral fatigue effects (Stutzig and Siebert, 2015; Stutzig and Siebert, 2016).

To further extrapolate the results of this study, future research should focus on combining different lifting techniques and combined movement tasks (e.g., lifting, carrying, placing) with varied lifting speeds. Combined with a more heterogeneous group, this could further expand the use of exoskeletons in occupational settings.

## 5 Conclusion

In this study, the commercially available active back-support exoskeleton Apogee (German Bionic Systems GmbH, Augsburg, Germany) was used to investigate the effect of an active exoskeleton on joint kinematics (ankle, knee, hip) and muscle activity (MVM, MBF, MES) during two different lifting speeds for a symmetric lift (stoop-technique). An increase in lifting speed led to a slight increase in muscle activity of the MBF and MVM, while MES activity remained unchanged. Increasing exoskeleton support reduced activity in the MES and MBF, accompanied by a modest increase in hip joint ROM.

These results suggest that the exoskeleton effectively supports the user even at higher lifting speeds, with no significant interaction between lifting speed and support level. This enables users to select the appropriate support level based on task requirements and personal comfort. In occupational settings, this flexibility could lead to a further expansion of exoskeleton usage with a potential reduction in development of MSDs.

## Data Availability

The raw data supporting the conclusions of this article will be made available by the authors, without undue reservation.
